# Epidermal growth factor receptor structural alterations in gastric cancer

**DOI:** 10.1186/1471-2407-8-10

**Published:** 2008-01-16

**Authors:** Cátia Moutinho, Ana R Mateus, Fernanda Milanezi, Fátima Carneiro, Raquel Seruca, Gianpaolo Suriano

**Affiliations:** 1 Instituto de Patologia e Imunologia Molecular da Universidade do Porto (IPATIMUP), 4200-465 Porto, Portugal; 2 Technische Universität München, Klinikum rechts der Isar, Institut für Allgemeine Pathologie und Pathologische Anatomie, D-81675 München, Germany; 3 Faculdade de Medicina da Universidade do Porto, 4200-465 Porto, Portugal

## Abstract

**Background:**

EGFR overexpression has been described in many human tumours including gastric cancer. In NSCLC patients somatic EGFR mutations, within the kinase domain of the protein, as well as gene amplification were associated with a good clinical response to EGFR inhibitors. In gastric tumours data concerning structural alterations of EGFR remains controversial. Given its possible therapeutic relevance, we aimed to determine the frequency and type of structural alterations of the *EGFR *gene in a series of primary gastric carcinomas.

**Methods:**

Direct sequencing of the kinase domain of the *EGFR *gene was performed in a series of 77 primary gastric carcinomas. FISH analysis was performed in 30 cases. Association studies between *EGFR *alterations and the clinical pathological features of the tumours were performed.

**Results:**

Within the 77 primary gastric carcinomas we found two *EGFR *somatic mutations and several *EGFR *polymorphisms in exon 20. Six different intronic sequence variants of *EGFR *were also found. Four gastric carcinomas showed balanced polysomy or *EGFR *gene amplification. We verified that gastric carcinoma with alterations of *EGFR *(somatic mutations or copy number variation) showed a significant increase of tumour size (*p *= 0.0094) in comparison to wild-type *EGFR *carcinomas.

**Conclusion:**

We demonstrate that *EGFR *structural alterations are rare in gastric carcinoma, but whenever present, it leads to tumour growth. We considered that searching for *EGFR *alterations in gastric cancer is likely to be clinically important in order to identify patients susceptible to respond to tyrosine kinase inhibitors.

## Background

Gastric cancer remains the second leading cause of cancer death worldwide [1] a scenario that highlights the need for more specific and efficient therapies. The exact mechanisms underlying gastric carcinogenesis are not yet fully understood, but evidence points to an association with pathways involved in developmental processes [2]. Key molecules of these pathways are the receptor tyrosine kinases (RTKs), which are found to be aberrantly activated or overexpressed in a variety of tumours and therefore represent promising targets for therapeutical intervention.

The members of the RTK superfamily of ERBB receptors are glycoproteins that consist of an extracellular domain where the binding of ligands takes place, a short lipophilic transmembrane domain, and an intracellular domain carrying the tyrosine kinase activity [3,4]. They are expressed in several tissues of epithelial, mesenchymal and neuronal origin, where they play pivotal roles in development, proliferation and differentiation. Deregulated expression of ERBB molecules, namely ERBB2, has been implicated in the development of numerous types of tumours, including gastric tumours. In gastric carcinoma it has been shown that ERBB2 overexpression is driven by gene amplification and is associated to carcinomas with high invasive potential [5]. ERBB1, better known as epidermal growth factor receptor (EGFR), overexpression has been described in many human tumours, including lung, colon, breast, prostate, brain, head and neck, thyroid, ovarian, bladder, kidney and also stomach cancer [6-11], and has been correlated to advanced tumour stage and poor clinical outcome. Very recently, we demonstrated that EGFR activation is associated to loss of function of E-cadherin, *in vitro *[12].

The mechanisms for oncogenic conversion of EGFR in cancer include amplified copy number, structural rearrangements of the receptor, and activating mutations [13]. EGFR mutations cluster in the kinase domain of EGFR (exons 18–21), and cause ligand-independent activation of the receptor, representing possible targets for therapeutical intervention. In this regard, somatic EGFR mutations as well as gene amplification in patients with non-small cell lung cancer (NSCLC) highly correlate with the clinical response to tyrosine kinase inhibitors [14,15].

In gastric tumours, data concerning structural alterations of EGFR remains controversial. Given its possible therapeutic relevance, in the present study we aimed to clarify the relevance of EGFR structural alterations in gastric carcinogenesis by analyzing a series of primary gastric carcinomas for copy number and mutations in the tyrosine kinase domain (exons 18–21) of the *EGFR *gene.

## Methods

### Case selection and histopathological classification of the tumours

Representative blocks of 77 formalin-fixed, paraffin embedded human gastric primary tumours were retrieved from the Department of Pathology of the Hospital S. João, after informed consent of the patients. Patients were informed that tumour material would be used for research purposes only. None of the patients included in the present series had a family history of gastric cancer. H&E- stained sections were used to categorize tumours according to the classifications of Lauren and Ming. Penetration of the gastric wall and the presence and localization of lymph node metastases were recorded for all patients using standard criteria for pathological staging. Orcein-stained sections were used for the detection of vascular invasion.

### EGFR Mutation Screening

Genomic DNA was extracted from 10 μm section after microdissection of the tumour areas to ensure a purity of at least 70% of neoplastic cells. DNA extraction was performed using the Genomic DNA Purification Kit (Gentra System) according to the manufacturer's protocol. Exon-specific primers were designed and DNA was subjected to PCR amplification of exons 18, 19, 20 and 21. The four *EGFR *exons code for the tyrosine kinase domain of EGFR. Primer sequences are shown in Table 1.

**Table 1:** Primers used for PCR amplification of the EGFR kinase domain

**Exon**		**Primer Sequence**	**PCR product size (bp)**
Exon 18	Forward	TGGGCCATGTCTGGCACTGC	283
	Reverse	ACAGCTTGCAAGGACTCTGG	
Exon 19	Forward	TCACTGGGCAGCATGTGGCA	241
	Reverse	CAGCTGCCAGACATGAGAAA	
Exon 20	Forward	CCTTCTGGCCACCATGCGAA	295
	Reverse	CGCATGTGAGGATCCTGGCT	
Exon 21	Forward	ATTCGGATGCAGAGCTTCTT	265
	Reverse	CCTGGTGTCAGGAAAATGCT	

PCR products were run on a 2% agarose gel and PCR amplified bands were extracted from the gel with the Gel Band Purification Kit (GE Healthcare). Samples were then purified and sequenced using the ABI Prism dGTP BigDye Terminator Ready Reaction Kit (Perkin Elmer, Foster City, CA) following manufacture's instruction and an ABI Prism 3100 Genetic Analyser (Perkin Elmer, Foster City, CA). The results were analysed using 3100 data collection software. Sequencing was performed in both strands. In cases with suspected mutations PCR amplification was repeated and the sample was re-sequenced to rule out PCR artefacts.

### EGFR Copy Number Variation Screening

The paraffin blocks were sectioned at 5 μm and tissue sections were dried at 60°C for 30 minutes. The slides were deparaffinised and washed followed by a pre-treatment and a digestion step using pepsin and finally dehydrated. The LSI EGFR Dual Color Probe-Hyb Set (VYSIS^®^), optimized to detect the band region 7p12 in spectrum orange and the centromere of chromosome 7 (7p11.1-q11.1, D7Z1 locus) in spectrum green, both in interphase nuclei and on metaphase chromosomes, was used. Five μl of the probe were applied to each slide. The denaturation was performed at 80°C for 8 minutes followed by hybridization on a humid chamber at 37°C for 16 hours. After hybridization, slides were washed and incubated with 4'-6-Diamidino-2-phenylindole (DAPI) for nuclear staining. For evaluation, sixty to 100 intact interphase nuclei were analysed by two independent observers in order to score the signals for the chromosome 7 centromere and the EGFR gene. Surrounding lymphocytes and normal mucosa were used as internal quality control for the assays. At least two or three representative areas of the neoplastic cells were selected, under a 100×/200× amplification field, to count the nuclei signals. After an overview under a 400× amplification, the signals were then counted using immersion oil (1000×).

### Statistical analysis

Association studies between EGFR alterations and the clinicopathological features of the cases were performed only in 30 cases (tumours analysed for EGFR mutations and EGFR copy number variation). Statistical analyses were assessed by the x2 test or Student's *t *test. A *p *value of <0.05 was considered statistically significant.

## Results

### EGFR Mutation Screening

**Figure 1:**
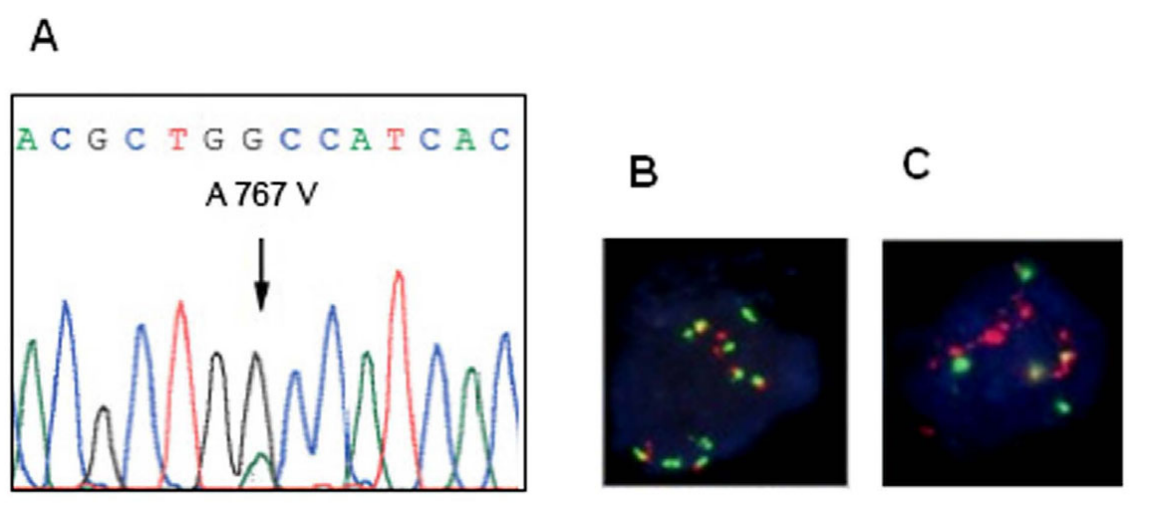
**Structural alterations in EGFR**. (A) Direct sequencing showing one of the mutations found in the kinase domain of EGFR – missense mutation (2300 C>T) in exon 20, leading to the substitution of the Alanine 767 for a Valine. (B) Diffuse gastric carcinoma with EGFR increased copy number caused by chromosome 7 polysomy. (C) Neoplastic cells exhibiting gene amplification with the formation of clusters with numerous signals for EGFR.

From the 77 gastric carcinomas analysed, *EGFR *mutations in exons 18–21 were detected in 2.6% (2/77) of the cases (Fig. 1A). One mutation belonged to exon 20 and was a missense mutation (2300 C>T) leading to the substitution of the Alanine 767 for a Valine. The second mutation affected exon 21 and was a missense mutation (2524 A>G) leading to the substituition of the Asparagine 842 for an Aspartic acid. None of the mutations were previously described. No sequence alterations were found in exons 18 and 19.

Several EGFR polymorphisms were found in exon 20 (Table 2). The polymorphism 2361G>A Gln787Gln, previously described by Mu *et al *[16], was present in 55.8% (43/77) of the cases, and in nine of the 43 cases in a homozygous state. We screened 50 normal controls and the 2361G>A Gln787Gln was present in 82% of the controls (41/50). Two other EGFR silent mutations (the 2301 C>T Ala767Ala and the 2415 C>T His805His) were found in exon 20, none of them previously described. Both alterations were found in a single case and were absent in normal controls.

**Table 2:** Sequence alterations found by direct sequencing

	**Alteration**	**Type**	**Frequency**	**References**
Exon 18				
	2184+19 G>A	Intronic variant	2/77	Ensembl SNP rs17337107 (dbSNP126) [17]
Exon 19				
	2185-9 C>G	Intronic variant	1/77	Not yet described
	2283+11 G>A	Intronic variant	1/77	Not yet described
	2283+47 G>A	Intronic variant	1/77	Not yet described
	2283+49 C>T	Intronic variant	1/77	Not yet described
Exon 20				
	2284-60 C>T	Intronic variant	2/77	Ensembl SNP rs10241451 (dbSNP126) [17]
	2300 C>T	Missense Ala 767 Val	1/77	Not yet described
	2301 C>T	Silent Ala 767 Ala	1/77	Not yet described
	2361G>A	Silent Gln 787 Gln	43/77	[16]
	2415 C>T	Silent His 805 His	1/77	Not yet described
Exon 21				
	2524 A>G	Missense Asn 842 Asp	1/77	Not yet described

We found six different sequence variants localized in intronic regions of EGFR, two of them previously described in Ensembl [17].

### EGFR Copy Number Variation Screening

The analysis of *EGFR *copy number, as assessed by fluorescence in situ hybridization (FISH), was only possible in 30 of the 77 cases analysed for EGFR mutations. All *EGFR *signals were compared to signals for centromeric probes for chromosome 7. More than 2.0 *EGFR *copies per cell (balanced polysomy or gene amplification) were detected in 13.3% (4/30) of the cases. Of the four cases showing more than 2 copies of *EGFR *per chromosome 7, three had increased copy number due to polysomy and one had gene amplification, a diffuse gastric carcinoma, exhibiting the formation of clusters with numerous signals for *EGFR *(Fig. 1B, C).

### EGFR structural alterations and clinicopathologic parameters of the patients and tumours

Table 3 shows the statistical associations between EGFR alterations and the clinicopathologic characteristics of the patients and tumours. When comparing gastric carcinoma harbouring EGFR alterations with carcinomas with normal EGFR status or copy number, we observed a significant association between EGFR structural alterations and increased tumour size. (*p *= 0.0094). No significant associations were found between EGFR alterations and other clinicopathologic parameters of the patients and tumours, namely gender and age of the patients, tumour localization, histological type, wall penetration, the presence of lymph node metastasis, vascular invasion and tumour staging of the tumour.

**Table 3:** Association of EGFR gene alterations with clinicopathological parameters

Clinicopathological parameters	**EGFR status**			
	
	Amplification/mutation (n = 6)	Normal (n = 24)	Total (n = 30)	*p *value
Sex (F/M)	1/5	10/14	11/19	0.2557
Age (SD)	62.3 ± 14,1	56.3 ± 16.8	57.5 ± 16.2	0.4271
Tumour localization				0.8548
Proximal	3	11	14	
Distal	3	13	16	
Size (SD)	11.6 ± 9.8	5.8 ± 2.0	6.9 ± 5.0	*0.0094
Lauren's classification				0.1261
Intestinal	1	12	13	
Diffuse	5	9	14	
Atypical	0	3	3	
Wall penetration				0.4642
Early (T1)	0	2	2	
Advanced (T2-T4)	6	22	28	
Vascular Invasion				0.7125
Absent (N0)	3	14	17	
Present (N = 1)	3	10	13	
Lymph node metastasis				0.1921
Absent	1	11	12	
Present	5	13	18	
Staging				0.3845
I	1	7	8	
II	0	5	5	
III	5	11	16	
IV	0	1	1	

## Discussion

EGFR gene is located on chromosome 7p12 and codes for a 170-KDa receptor, present in the membrane of cells as an inactive monomers. Upon ligand binding to the extracellular domain, the receptor undergoes conformational changes, dimerises and becomes autophosphorylated in key tyrosine residues in the intracellular tyrosine kinase (TK) domain. This leads to the activation of downstream pathways which control cell survival, inhibition of apoptosis and proliferation [18].

Much attention has been drawn to the oncogenic effect of EGFR and most of all to success of EGFR target therapies, which are well established for non small cell lung carcinomas. The role of EGFR in gastric cancer is very controversial. Some authors reported that EGFR is highly expressed in gastric cancer, suggesting its suitability as a target for receptor tyrosine kinase inhibitors [19,20]. Conversely, Takehana and colleagues reported that overexpression of epidermal growth factor receptor is a rare event in gastric carcinoma [21] and occurs predominantly due to EGFR gene amplification, confirming results from previous studies [22-24]. EGFR mutations in primary gastric carcinoma or gastric cancer cell lines were never reported [25-27]; nevertheless we recently showed that hereditary diffuse gastric cancer associated E-cadherin germline missense mutations lead to increased EGFR activity.

On this basis, we considered that searching for EGFR alterations in gastric cancer might have been important to identify therapy susceptible cases.

Here we report the presence of *EGFR *increased copy number in 13.3% of the 30 cases in which FISH analysis was possible. Of the four cases with increased copy number, just one presented gene amplification, whereas the remaining 3 cases showed polysomy of chromosome 7. These results match previous reports investigating the EGFR copy number in gastric cancer [22-24]. The mutational analysis of the kinase domain of EGFR revealed the presence of mutations in 2.6% of 77 gastric carcinomas. The identified mutations were of the missense type and were present in exons 20 and 21 of the EGFR gene. None of the identified mutations had been previously described and their functional significance is not yet assessed. However, due to their localization in the kinase domain of EGFR, it is tempting to speculate that they affect the activity of the receptor and therefore patients harbouring these EGFR mutations may benefit from tyrosine kinase inhibitors as therapeutic approach.

Besides these mutations found, other sequence alterations were identified, all clustering in exon 20. The EGFR polymorphism 2361 G>A (previously described in Ensembl) occurs in a high percentage of cases and in normal controls. In contrast, the other two not yet described silent variants in exon 20 (2301 C>T, 2415 C>T) were absent in normal controls. In addition to these EGFR sequence variants in coding regions, we have also identified variations in the intronic sequence flanking exons 18, 19 and 20, but their functional effect remains unclear.

The correlation of the clinical parameters for the cases for which both mutation and FISH analysis was possible, showed a significant association between EGFR alterations and tumour size. Interestingly, all cases with alterations in EGFR (amplification/mutation) were carcinomas already invading the basal membrane and spreading into the gastric wall (T2-T4), suggesting that alterations of this gene may confer an invasive behaviour to neoplastic cells.

However, this hypothesis needs to be clarified by further studies, since we did not verify a statistically significant correlation between EGFR alterations and invasion in the gastric wall (p = 0.4642), as we have just analised a very low number of early gastric carcinomas (n = 2) in our series. Both results are in agreement with the reports of Hirono *et al*., 1995 [23] and Tsugawa *et al*., 1998 [24], suggesting that EGFR is involved in tumour growth and activating alterations may be a late event involved in tumour progression.

Although no other statistically significant association was found, it is interesting to notice that EGFR alterations occur mainly in carcinomas of the diffuse type. The presence of EGFR alterations in diffuse carcinomas is in contrast to what previously observed for other members of the ERBB receptor family in gastric cancer. In gastric carcinoma ERBB2 amplification was detected preferentially in intestinal carcinomas of the stomach [5].

## Conclusion

In conclusion, in this study we demonstrated that EGFR activating alterations are not a frequent event in gastric carcinogenesis. Alterations preferentially appear in gastric carcinomas of the diffuse subtype and are associated to tumour size. Despite the low frequency of EGFR alterations, our results also indicate that there is a restricted group of selected gastric patients that might benefit from non conventional therapies, including pharmacological inhibitors of the EGFR receptor.

## Competing interests

The author(s) declare that they have no competing interests.

## Authors' contributions

RS and GS were responsible for study concept and design, as well as study supervision. CM carried out the molecular genetic studies and participated in the drafting of the manuscript. FM and FC were responsible for analysis and interpretation of data. ARM collected the data and performed the drafting of the manuscript. RS and GS did the critical revision of the manuscript for important intellectual content. All authors read and approved the final manuscript.

## Pre-publication history

The pre-publication history for this paper can be accessed here:


